# 

**Published:** 1873-01

**Authors:** 


### INDEX FOR 1873.—VOL. XX.

PAGE.

A New Leaf ... 1
Athletic Sports ... 17
Air, Pure ... 142
Absence of Editor ... 168
Age, Old Desirable ... 235

Bad Temper ... 16
Bare Limbs ... 31
Bruises, ... 33
Breath, Sweet ... 34
Bitters, Bad ... 75
Burned to Death ... 78
Business Failures ... 85
Boys, Poor ... 86
Breed, Coarse ... 130
Benefactors ... 147
Baby Treatment ... 156
Boils, ... 186
Back Bone ... 207
Bed Chambers ... 242
Be Agreeable ... 262

Chillblains ... 12
Coal Curiosities ... 26, 280
Children Raising ... 34, 264
Clergymen ... 49, 60, 274
Catarrh ... 51, 243
Communism ... 55
Changing Clothing ... 75
Cold Feet ... 83
Cement, ... 86
Chlosel ... 90
Consumptives ... 91, 108, 247
Costiveness ... 92
Checking Perspiration ... 94, 164
Corns ... 103
Chimneys, Smoky ... 141
Calomel ... 143
Cellars ... 157
Condiments ... 181
Castor Oil ... 185
Children Fretful ... 222
Children's Diseases ... 223
Cromwell ... 234
College Days ... 241
Christianity Aggressive ... 265
Christmas ... 267

Dandruff ... 52
Dangers, Four ... 211
Death, Meeting ... 29
Diptheria ... 30
Dyspeptic ... 213
Death Tests ... 81
Developers ... 88
Disinfectants ... 88
Divorce ... 125

PAGE

Drowning ... 138
Digestion ... 143
Death Rate ... 161
Drinking Water ... 164
Death Weapons ... 167
Drunkards ... 184

Earning a Living ... 9
Eating too Fast ... 79
Eating wisely ... 93
Eating and Drinking ... 129
Editor's Absence ... 168, 225
Eating Regularly ... 238
Eloquence, Secret of ... 265, 279

Frost Bite ... 13
Flowers and Paintings ... 14
Foreign Travel ... 24, 97
Fat Meat ... 40
Fire on the Hearth ... 76
Fidelity ... 186
Family Reading ... 193
Fixed Faith ... 201
Flannel Shirts ... 272
Fruit, Dried ... 84
Failures, Business ... 85
Fomentations ... 87
"Funning" us ... 88
Feet, Cold ... 83, 266
Flannel, Wearing ... 91

Girls, Homeless ... 10
Getting Angry ... 16
Games ... 17
"Good Things" ... 44
Going Abroad ... 97
Good, Doing ... 151
Going up Stairs ... 196
Gentleman, A ... 198
Going to College ... 241

Home, A Cheerful ... 7
Hair Dyes ... 19
Hay Fever ... 28
Horses and Men ... 45
Homes for Emigrants ... 71
Health Essentials ... 91
Honest Quaker ... 105
Horse Doctors ... 114
"Health at Home" ... 121
Hard Study ... 177
Household Knowledge ... 188
Hospitals ... 192
Health Principles ... 239
"Helping Hand," The ... 271

Ice Houses ... 42

PAGE.
Investing Safely.......................... 103
Irritating.......................... 126
Insanity.......................... 133
Illustrations.......................... 143
Ill Nature.......................... 257

Jail Cure.......................... 89
Josh Billings.......................... 90
Jew and Gentile.......................... 199

Leaf, A New.......................... 1
Lead Pipes.......................... 28
Living Well.......................... 39
Lamp Shades.......................... 53
Letter Writing.......................... 56
Lent.......................... 74
Longevity.......................... 87
Lenox, The.......................... 107
Letters from Abroad.......................... 203
Lungs Measurer.......................... 247
Losing Children.......................... 264

Man's Make up.......................... 5
Music..........................11, 263
Manners, Two..........................29, 261
Matches, Friction.......................... 43
Men and Horses.......................... 45
Medicines, Good.......................... 58
Mothers, Young.......................... 166
Man's Rights.......................... 187

Nursing, Scientific.......................... 136
Nose Bleed.......................... 140

Oatmeal.......................... 132
Ornaments, Poisonous.......................... 142
Omnibus.......................... 209
Old Age.......................... 215

Pay as You Go.......................... 4
Paintings.......................... 14
Plays and Games.......................... 17
Parental Influence.......................... 25
Preacher, Old, The.......................... 32
Pickles.......................... 67
Painful Affections.......................... 80
Poor Boys.......................... 86
Pomage.......................... 132
Poisonous Ornaments.......................... 142
Pure Air.......................... 142
Pork Worm.......................... 159
Physicians.......................... 181
Puzzles.......................... 191
Preaching, Paris.......................... 277
Pews.......................... 227
Pity the Poet.......................... 272
Plants in Chambers.......................... 278
Public Speaking.......................... 279

Quinine.......................... 185

Rat Riddance.......................... 34
Red Ants.......................... 44
Reading Good.......................... 82
Riches with Wings.......................... 103
Railroad Travel.......................... 109
Respiring.......................... 126
Rouse Up.......................... 135

Skating.......................... 7
Summer Drinks..........................31, 111
Sweet Breath.......................... 34
Stupidities.......................... 53
Students, Theological.......................... 60
Sing More.......................... 63
Spring Dangers.......................... 73
Sleeping Badly.......................... 81
Safety Lamps.......................... 89
Summer Hues.......................... 111
Shoes in Summer.......................... 111
Sick, Where to be.......................... 137
Smoky Chimneys.......................... 141
Summer Complaint.......................... 153
Stimulants.......................... 157
Sick Rooms.......................... 183
Spine.......................... 207
Suicide Prevented.......................... 222
Style.......................... 263
Sleeping with Plants.......................... 278
Society, the Best.......................... 272

Teeth Drawing.......................... 69
Tuke, William.......................... 134
Trichiniæ.......................... 159
Temper on Health.......................... 264

Useful Items.......................... 190
Unappreciated.......................... 273

Vaccination.......................... 197
Vital Capacity.......................... 248

Wife, A Good.......................... 20
Winter Wisdom..........................21, 41
Wounds and Bruises.......................... 33
Which the Wiser?.......................... 34
Wall Paper.......................... 45
Workers, the World's.......................... 49
Work and Study.......................... 60
Wearing Flannel.......................... 91
Where be Sick.......................... 137
Waters, Unhealthy.......................... 141
Water for Drinking.......................... 164
Weapons of Death.......................... 167
Work and Works.......................... 177
Wag, Worth of a.......................... 178
Whitlows.......................... 186
Weights and Measures.......................... 190
Wealth and Sense.......................... 259

Young Mothers.......................... 166

